# Performance of automated slidemakers and stainers in a working laboratory environment – routine operation and quality control

**DOI:** 10.1111/j.1751-553X.2009.01141.x

**Published:** 2010-02

**Authors:** E SIMSON, M G GASCON-LEMA, D L BROWN

**Affiliations:** Hematology Laboratory, Department of Pathology, Mount Sinai Medical CenterNew York, NY, USA

**Keywords:** Peripheral blood films, quality control, automated slidemaker/stainers, leukocyte differential counts, blood cell morphology

## Abstract

The automated slidemaker/stainers of the four Beckman Coulter LH755 hematology systems in our laboratory are operated as analyzers, with similar requirements for setup, maintenance and quality control. A study was performed to confirm that these slide maker/stainers in routine use produce peripheral blood films that are completely satisfactory for microscopy and without cells, particularly abnormal cells, being pulled to the edges or sides of the film outside the usual working area. One hundred and thirty-nine automated blood films that had been produced during routine operation were compared with well-prepared manual films from the same patients. None of the films was unacceptable for microscopy. The distributions of normal white cell types within the counting areas of automated films compared with manual films, for all 139 samples for WBC from 1.0 to 352.8 × 10^9^/l; for blasts and promyelocytes in the 65 samples in which they occurred and for nucleated red blood cells in the 58 samples in which they occurred all fell within the expected limits of 200 cell differential counts of CLSI H20-A. Red cell morphology and the occurrence of WBC clumps, platelet clumps and smudge cells were comparable between the automated and manual films of all samples. We conclude that automated slidemaker/stainers, as typified by those of the Beckman Coulter LH755 system, are capable of producing blood films comparable with well-prepared manual films in routine laboratory use; and that the maintenance and quality control procedures used in our laboratory ensure consistent high quality performance from these systems.

## Introduction

The expanded capabilities of multi-channel hematology analyzers have resulted in fewer films being examined by visual microscopy, especially after criteria, such as the International Consensus Group criteria (Barnes *et al.*, 2005) are applied to results from hematology analyzers. The fewer films that are examined consist primarily of abnormal samples, where the requirement for each of the films to be of good quality is greater. As hematology laboratories have become more automated, the use of automated slide makers and stainers has increased. Performance characteristics of these systems are not well known, because the literature is extremely lacking in studies of automated slidemaker/stainer performance and also because laboratories usually place these instruments in use without formal performance studies other than confirming satisfactory mechanical operation. It is not well known whether the automated blood films are satisfactory for microscopy, without distortion of cell morphology and without cell clumping or other interference; and whether any cells, particularly abnormal cells in a variety of diseases, may be pulled to the edges or sides of the film outside the usual working area. It is well known that cells, especially monocytes and other large cells, may be pulled to the edges or sides of the film if there is maldistribution of leukocytes in the blood film. It is obviously more important to detect and count abnormal cells, particularly if they occur in low frequency. Automated slidemaker/stainers have been used routinely in our laboratory for several years to produce satisfactory blood films, beginning with two predecessor Coulter GenS systems (Beckman Coulter Inc., Miami, FL, USA). The automated slidemaker/stainers of the four Beckman Coulter LH755 hematology systems (Beckman Coulter Inc.) in our laboratory are operated as analyzers, with similar requirements for setup, maintenance and quality control. Many visitors to our highly automated core laboratory have admired the quality of the blood films, including the stain, produced by our slidemaker/stainers and have asked for copies of our procedures. We have also heard anecdotally, without having been informed of specific data, of other slidemakers that might not be performing as well as ours. With four slidemaker/stainers in routine use, we felt there was a good opportunity to collect data on multiple instruments in a real-life laboratory situation on a wide variety of patient samples; make our procedures, particularly for quality control and including staining more widely available; and add useful information to the literature. We performed a study using CLSI Standard H20-A (Koepke *et al*. 1992) for the Reference Leukocyte Proportional Differential Count to compare the performance of automated films with well prepared manual films.

## Methods and materials

A total of 139 patient samples with a wide range of WBC counts and abnormal cells were selected from the daily routine workload of the laboratory. Samples for this study were selected for the presence of a number of different abnormal cells in a variety of diseases as well as diseases that might interfere with the production of blood films of good quality. Samples were selected for the study after the sample had been analyzed and the automated blood film had been produced. These samples were processed on 41 days over a period of several months. An automated slide had been prepared from each sample as part of the routine analysis of that sample with any of the four Beckman Coulter LH755 systems in routine laboratory use. In no instance was a sample re-run on a slidemaker/stainer to produce a better quality blood film than that obtained when the sample was run for the first time. The Beckman Coulter LH755 instrument consists of an LH750 multiparameter hematology analyzer, a slidemaker module and a stainer module. A summary of our settings and maintenance procedures for the slidemaker/stainers is shown in Table 1. The procedures for setup and maintenance followed manufacturer instructions, as found in both the operator’s manual (COULTER®, 2006) and the ‘help’ screens of the analyzer systems’s data workstation. We made some modifications to the staining procedure as summarized in Table 2 and detailed in Appendix 1. Daily quality control procedures are shown in Table 3. A manual film was made from each sample following the procedure of CLSI H20-A and stained by manual feeding of the stainer module of the Beckman Coulter LH755. In contrast to H20-A, only one slide was produced for each of the manual and automated slide preparations instead of three slides of each, because of operational constraints in a large and very busy hematology laboratory.

**Table 3:** Quality control for slidemaker/stainers

At startup of each LH755 system every morning
Probe and rinse cup on each slidemaker/stainer are inspected and cleaned if necessary
Two films are made from patient samples
Each film is examined macroscopically and microscopically for quality of film and
stain
Approximately 2.5 cm long, terminating 1 cm from edge
Gradual transition in thickness from thick to thin areas
No grainy streaks, troughs or ridges and no artifacts
Narrower than width of slide, with smooth edges
Adequate working area with acceptable morphology
Minimal cellular distributional distortion
Nuclei and cytoplasm of different cell types stained satisfactorily

**Table 2:** Stain procedure modifications

Added quick rinse phase (1 : 5 methanol : water)
Reduced stain time from 15 to 7 min
3–4 min drying time

**Table 1:** Slidemaker/stainer setup

Glass slides*
Precleaned (polished) on both surfaces
Beveled edges with one frosted end
No cracks or chips
Uniform thickness
Same slides used throughout laboratoryfor all blood films, so as to avoid inadvertentuse of inferior quality slides on the slidemakers
Slidemaker/stainer
Spreader pressure carefully adjusted
Drop size: 2 (settings range 1–4)
Wick time: 6 (settings range 1–10)
Wick speed: 6 (settings range 1–10)
Maintenance
Stain baths drained and refilled daily
Stain bottle changed weekly
Baths and trays cleaned monthly

* We use S/P Brand Bev-L-Edge Micro Slides (Cat. M6167-2; Allegiance Healthcare Corporation, McGaw Park, IL, USA). Frosted at one end, both sides and 1.0 mm thick, precleaned, size 3” × 1”.

Two senior technologists in our laboratory, each with several years of experience in performing microscopic morphological blood film review and visual WBC differential counts, performed morphological film review and 200 cell WBC differential counts on the automated films and manually prepared films from each sample according to CLSI Standard H20-A. The two microscopists were instructed to perform 200 cell leukocyte differential counts as per CLSI H20-A, to assess red cell morphology and in addition to comment on any observed artifacts and film quality. Each observer reviewed and performed counts on 278 films, consisting of 139 automated and 139 manual films. Their individual results were reviewed separately for consistency and then combined to produce an averaged 200 cell count for each automated and each manual film. Because the same films were examined by both observers, it is very likely that the majority of the cells examined by the microscopists were the same, so that the cell count was not a total 400 cell count, but rather a duplicate 200 cell count by two independent observers to minimize interobserver variability in cell identification. Each observer was unaware of the results of the other observer for each sample and of his/her own results for the other slide of the automated/manual pair. The statistical envelopes for imprecision of 200 cell counts were derived from the Standard Error of Proportion calculations of H20-A.

## Results

All samples were from adult patients, as most pediatric sample tubes are too small to be placed on our automated system. As shown in Table 4, the samples studied included 31 samples from normal patients; 75 samples from patients with a variety of malignant diseases, both new cases and patients undergoing treatment; three with benign lymphocytosis; one with eosinophilia; 10 with acute bacterial infection; seven with renal disease on dialysis; six with hemoglobinopathies; and six with a variety of abnormal chemistry results, mainly hyperlipidemia. The malignant diseases included acute myeloid leukemia (AML), B- and T-cell acute lymphocytic leukemia (ALL), precursor B-lymphoblastic ALL, biphenotypic myeloid and T-cell acute leukemia, acute leukemia not further specified, blastoid phenomorphic variants of mantle cell lymphoma, NK lymphoma, Hodgkin’s lymphoma, chronic lymphocytic leukemia (CLL), myelodysplasia, myelodysplasia transforming to AML and multiple myeloma.

**Table 4:** Total of 139 patient samples

Diagnosis	*n*
Normal	31
AML	28
Myelodysplasia	12
Lymphoma	11
B cell ALL	6
Acute leukemia	8
CLL	3
Myeloma	7
Abnormal hemoglobin	6
Lymphocytosis	3
Acute infection	10
Dialysis	7
Elevated chemistries	6
Eosinophilia	1

CLL, chronic lymphocytic leukemia; ALL, acute lymphocytic leukemia.

The WBC counts ranged from 1.0 to 352.8 × 10^9^/l. The WBC counts were distributed as follows: 23 samples had WBC between 1.0 and 5.0 × 10^9^/l; 51 had WBC between 5.1 and 10.0 × 10^9^/l, 33 had WBC between 10.1 and 20 × 10^9^/l, 15 between 20.1 and 50 × 10^9^/l; 11 between 50.1 and 100 × 10^9^/l; five between 100.1 and 200 × 10^9^/l; and there was a CLL with a WBC of 352.8 × 10^9^/l.

**Figure 1:**
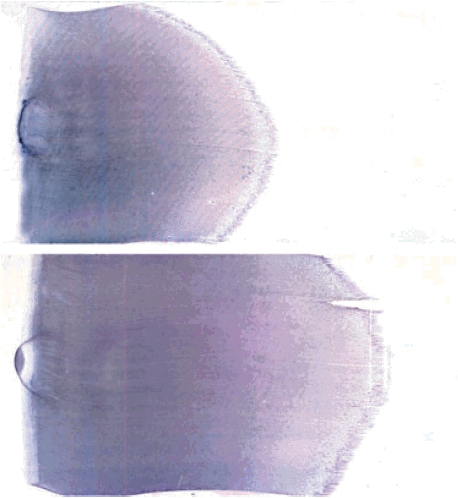
Examples of manually prepared blood films.

**Figure 2:**
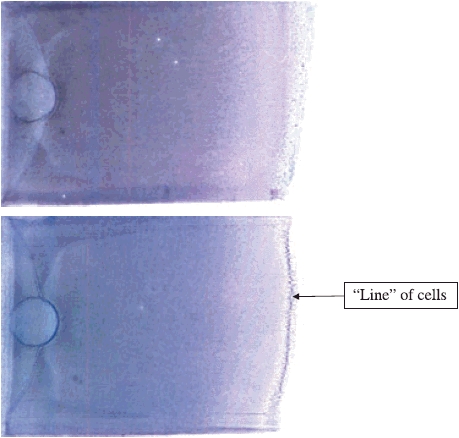
Examples of blood films produced by the automated slidemaker/stainers. The second blood film shows the ‘line’ of cells in the tail observed on a few of the automated blood films.

Macroscopically, the automated blood films were of good quality, with a gradual transition in thickness, smooth continuous side margins and the other desirable qualities of a blood film as described in CLSI H20-A. Photographs of representative smears made manually are shown in Figure 1 and of blood films produced by the automated smearmaker/stainers are shown in Figure 2. A few of the automated blood films were observed to have a ‘line’ of cells at the tail of the film, as shown in Figure 2. No blood films were judged unacceptable for morphological review and differential counting.

**Figure 3:**
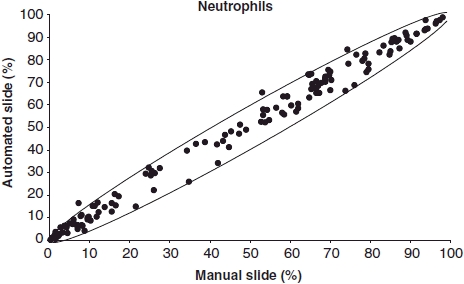
Comparison of neutrophil counts from automated slides with neutrophil counts from manual slides. Points represent averages of 200 cell counts by two observers on 139 patient samples. Lines represent the 95% confidence envelope for imprecision of 200 cell differential counts from CLSI H20-A.

**Figure 4:**
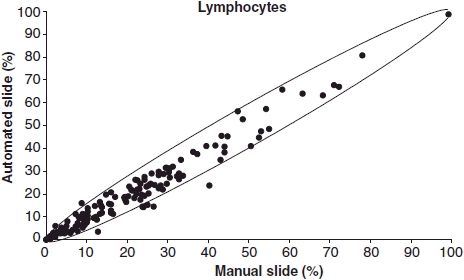
Comparison of lymphocyte counts from automated slides with lymphocyte counts from manual slides. Points represent averages of 200 cell counts by two observers on 139 patient samples. Lines represent the 95% confidence envelope for imprecision of 200 cell differential counts from CLSI H20-A.

**Figure 5:**
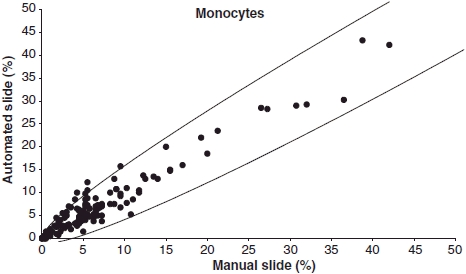
Comparison of monocyte counts from automated slides with monocyte counts from manual slides. Points represent averages of 200 cell counts by two observers on 139 patient samples. Lines represent the 95% confidence envelope for imprecision of 200 cell differential counts from CLSI H20-A.

**Figure 6:**
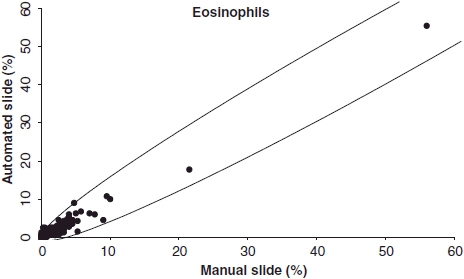
Comparison of eosinophil counts from automated slides with eosinophil counts from manual slides. Points represent averages of 200 cell counts by two observers on 139 patient samples. Lines represent the 95% confidence envelope for imprecision of 200 cell differential counts from CLSI H20-A.

**Figure 7:**
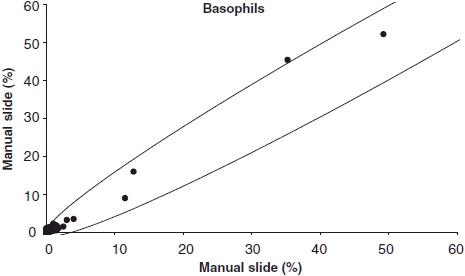
Comparison of basophil counts from automated slides with basophil counts from manual slides. Points represent averages of 200 cell counts by two observers on 139 patient samples. Lines represent the 95% confidence envelope for imprecision of 200 cell differential counts from CLSI H20-A.

The distributions of normal white cell types within the counting areas of automated films compared with manual films, for all the WBC from 1.0 to 352.8 × 10^9^/l fell within the expected limits of 200 cell differential counts as determined by the Standard Error of Proportion calculations and graphs of CLSI H20-A. In this analysis, which is based on 95% statistical confidence for imprecision, it is expected that up to 5% of the results would be outside the expected limits. The actual percentages outside the expected limits were: neutrophils 3.6% (five of 139 samples), lymphocytes 4.3% (six samples), monocytes 3.6% (five samples), eosinophils 2.9% (four samples) and basophils 2.2% (three samples). The graphs for neutrophils, lymphocytes, monocytes, eosinophils and basophils are shown in Figures 3–7.

**Figure 8:**
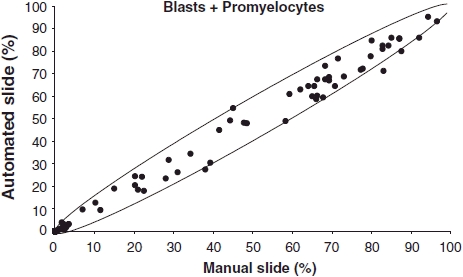
Comparison of counts of blasts and promyelocytes between automated slides and manual slides. Points represent averages of 200 cell counts by two observers on 65 patient samples. Lines represent the 95% confidence envelope for imprecision of 200 cell differential counts from CLSI H20-A.

Blasts were found in a total of 58 samples; promyelocytes were found together with blasts in 10 of these samples and in seven samples without blasts. The distributions for blasts and promyelocytes in the 65 samples in which they occurred and for nucleated red blood cells (NRBC) in the 58 samples in which they occurred also fell within the expected limits of 200 cell differential counts of CLSI H20-A, as shown in Figures 8 and 9. The actual percentage outside the expected limits for blasts and promyelocytes was 4.6% (three samples) and for NRBC was 1.7% (one sample). Counts for a variety of abnormal cells for proportions as low as 1% were similar on automated and manual films; no sample with abnormal cells was ‘missed’ on any automated or manual film.

**Figure:**
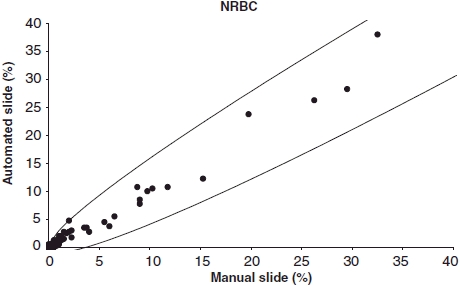
Comparison of NRBC counts from automated slides with NRBC counts from manual slides. Points represent averages of 200 cell counts by two observers on 58 patient samples. Lines represent the 95% confidence envelope for imprecision of 200 cell differential counts from CLSI H20-A.

The presence of smudge cells in decreasing order of frequency was noted as follows: 3/high-power field (HPF); 2/HPF; 1/HPF; <1/HPF or present in small numbers; and occasional or rare. There was a total of 19 samples where smudge cells were observed on both the automated and manual blood films. Smudge cells were primarily seen on neoplastic samples. The CLL sample with the WBC of 352.8 × 10^9^/l and two samples from patients with NK cell leukemia/lymphoma and white cell counts of 66.3 and 58 × 10^9^/l had three smudge cells/HPF. Two smudge cells/HPF were observed in a patient with myelodysplasia and a WBC of 90.7 × 10^9^/l. One smudge cell/HPF was seen in seven patients with AML, Hodgkin’s lymphoma, B precursor ALL, and NK lymphoma. Less than one smudge cell/HPF or the presence of smudge cells in small numbers was seen in four patients with myelodysplasia and AML. Occasional or rare smudge cells were seen in patients with acute leukemia and mantle cell lymphoma; and one patient with an increased serum lactic dehydrogenase concentration without a further specific diagnosis.

In addition, rare or occasional smudge cells were observed on six automated films but not on the manual films in a patient with sepsis, an acute leukemia, a benign lymphocytosis, increased cholesterol and uric acid, increased protein, and a normal patient.

Auer rods were observed in the blasts of both the automated and manual films of five samples. Toxic granulation was observed in the neutrophils on both the automated and manual films of 14 samples, in one sample on the automated film only and in three samples on the manual film only. In three samples, Pelger-Huet cells were observed on both automated as well as manual films. One case was a hairy cell leukemia; the characteristic cytoplasmic projections were seen on both automated as well as manual films.

A ‘line’ of cells was observed at the tail of the film on a few of the automated films, as shown in Figure 2. No differences in cell counts between the automated and manual films obtained from the counting area occurred in these samples. WBC clumps, defined as two or three cells touching each other, were observed on the sides of the film on 13 (9.3%) of the automated slides and 11 (7.9%) of the manual slides. WBC clumps were observed in the tail of the film on 40 (28.0%) of the automated slides, including those with the ‘line’ of cells and 23 (16.5%) in the tail of the film of the manual slides. We do not consider clumps of two or three cells to be significant morphologically. Morphologically significant increased WBC clumps, defined as four or more cells in the clump, occurred on the side of the film in one each of the automated and manual slides; and in the tail of the film in four (3%) of the automated slides and two (1.5%) of the manual slides.

Red cell morphology was comparable between the automated and manual films of all samples and was consistent with the diagnosis. Occasional platelet clumps were observed on both the automated and manual films of three samples.

## Discussion

### Role of blood film examination in the modern automated hematology laboratory

From the time of the first automated hematology analyzer in 1954 invented by Wallace Coulter (Coulter, 1953), through the further development of multiparameter hematology analyzers including those capable of performing an automated flow differential count (Mansberg, Saunders & Groner, 1974) to the present time, the capabilities of these analyzers have become progressively more comprehensive. Automated hematology analyzers produce superior red cell, white cell and platelet counts compared with manual methods. The analyzer flow type WBC differential count is superior to the visual microscopic film differential count for mature white cell types (Simson & Groner, 1995). Modern multi-parameter hematology analyzers also provide flagging for a number of white cell, red cell and platelet morphological abnormalities. However, visual examination by microscopy of a blood film on a glass slide remains an essential component of the complete hematology analysis of peripheral blood samples for selected samples, especially those for which the analyzer has flagged the presence of abnormal cells that cannot be satisfactorily counted by the analyzer.

In the modern hematology laboratory, for reasons of efficiency, cost-effectiveness and the combination of the best aspects of analyzer capabilities with human capabilities, criteria for action after hematology analyzer results are commonly used. An International Consensus Group published guidelines to assist laboratories in establishing criteria for action, including result reporting without further review (Barnes *et al.*, 2005). The most common follow up action is microscopic visual review of a peripheral blood film and blood films are made only from those samples meeting criteria for microscopic review. Using review criteria, blood film review rates vary from approximately 10% to 50% in different laboratories (Novis *et al.*, 2006). With this selective production of blood films, the quality of every film is more important than if films were made from every sample, where some poor films might not even have to be examined. Highly automated laboratories are using automated slidemaker/stainers to produce slides for microscopic review of peripheral blood films. In our laboratory, the slidemaker module and the stainer module are integrated with the analyzer in one instrument. Criteria programmed into the hematology analyzer instruct the slidemaker/stainer modules to make and stain a film based on the results of that analyzer’s analysis of that sample at that time.

### Characteristics of blood films and adjustments of automated smearmaker/stainers

Slidemakers are mechanical devices that typically reproduce the manual wedge film procedure for making a blood film. The slidemaker/stainers of the Beckman Coulter LH755 systems in our laboratory have adjustable primary settings for blood drop size, wick time, wick speed and pressure of the spreader slide on the object glass slide; together with the ability to automatically change the angle of the spreader slide based on the hematocrit of each sample. As mechanical devices slidemaker/stainers have the potential to make films more consistently and reproducibly than the manual wedge method, provided that they have been set up correctly and that the glass slides used are of high quality, free of grease and of uniform thickness. For example, if the pressure of the spreader slide on the object slide is too low, blood from the drop can flow forward of the spreader slide edge and be pushed at the front edge of the film rather than pulled behind the spreader slide edge, with resultant white cell distributional distortions as well as morphological inadequacy of the working area. Alternatively, if the pressure is too great, the spreader slide can skip along the object slide and the film will not be smooth, also resulting in distortions. Differences in thickness from slide to slide can play havoc with the most careful spreader slide pressure settings.

### Staining of blood films

Stain quality is an important component of a satisfactory film for visual examination by microscopy. The Romanowsky effect can be obtained by several different combinations of staining materials. CLSI has published a standard for Romanowsky stains (Marshall *et al.* 1986). The manufacturer of the stainer module supplied with our system has a recommended stain and recommended staining procedure, but makes provision for the user to alter stain and wash times as well as the ability to use different stains than those supplied. We use the supplied stains, but have modified the preparation of the reagents as well as the staining procedure. We have found that this provides an enhanced stain for microscopy, as well as reducing the staining time by several minutes to improve rapidity of film examination and reporting. This modified staining procedure is summarized in Table 3 and detailed in Appendix 1.

### Quality control of smearmaker/stainers

Laboratory maintenance procedures were performed according to the manufacturer’s instructions. Settings for spreader slide pressure, drop size, wick time and wick speed must be established by trial and error, but in our experience, once set, they produce films of consistent quality for each sample for long periods of time. However, our laboratory is in operation 24 h/day, 7 days/week. There are three shifts per day with different staff on each of the shifts and different weekend staff. The four slidemaker/stainers are all in routine daily operation. The slidemaker settings may need to be changed over time and the settings may be changed from time to time in response to perceived problems. It is necessary to have a method of quality control to assure any operators who make adjustments to the settings that these adjustments have resulted in films of good quality. We have instituted daily quality control by making two films from patient samples on each of the four slidemaker/stainers early during the morning shift and examining these macroscopically and microscopically for quality of the film. We operate the slidemaker/stainers in the same way as any analyzer in the laboratory, whereby if the quality control results are unsatisfactory, the analyzer is not used for patient samples until the problem has been corrected. If quality control films from any of the slidemaker/stainer units are unsatisfactory, that device is checked and cleaned or re-set as necessary to produce satisfactory films before being used for patient samples. In our experience, it is infrequent that the films are unsatisfactory, but we do have the assurance on a daily basis that all slidemaker/stainers are operating properly. In addition, the quality control process focuses technologist attention on the need to maintain the devices properly and assure satisfactory slides.

### Issues in assessment of comparability between automated and manual blood films

#### Blood film characteristics

Assessment of comparability between automated and manual films is complicated by the distortions of morphology produced inherently by the process of making a film, whereby shear forces are applied to the cells to separate them in a more or less even fashion in a large enough area of the film to allow for good visualization microscopically. Cells are flattened in this process, so that, e.g. lymphocytes that are spheroid *in vivo* appear as discs with a relatively central nucleus and with a thin rim of cytoplasm; neutrophils that *in vivo* are motile with morphological alterations as they move appear also as flattened discs with some irregularity of cell borders and with segmented nuclei. Red cells that *in vivo* are biconcave discoid envelopes with significant thickness toward the outer areas of the cells appear as discs with only a central pallor to suggest the thinner central portion of the cell. It is well known that a large portion of the film extending from the point where the drop of blood has been applied is thick and has too many overlapping cells of all types for adequate morphology. The tail of the film contains red cells that have been too stretched by shear forces to permit good assessment of red cell morphology and also contains white cells with poor morphology that may occur in small clumps. The acceptable ‘working area’ of the film extends from the area where approximately 50% of the red cells overlap to the area where the red cells show a strong tendency to linear orientation, as detailed in CLSI H20-A.

#### Precision of the differential count

Assessment of comparability between automated and manual films is complicated by the imprecision induced by counting a relatively small number of cells in the microscopic WBC differential count, typically 100 white cells in the routine count. What may appear to be large differences between counts can occur purely because of the statistical limitations of the proportional count of a small number of cells. When white cells occur with low frequency, e.g. 2–3% of blasts, a 100 cell differential count may not include them, purely on statistical grounds, with the resultant impression that the observer has ‘missed’ a significant abnormality, or that the slidemaking procedure has removed these cells from the film. Interobserver variability is also known to cause differences in results from visual differential counts. CLSI H20-A attempts to reduce imprecision (and also interobserver variability) by having two trained observers count a total of 400 cells from a minimum of two films from each sample, but even this increased count has inherently large imprecision compared with analyzers that typically count several thousand white cells to obtain the differential count. For this reason, H20-A has been shown to be inadequate for adequately assessing performance of analyzer differential counting (Simson & Groner, 1995). However, despite its limitations for analyzer performance, H20-A provides a good method for comparing visual differential counts, so its principles were followed to compare visual differential counts on slides produced by the two different methods in this study. In our study, in contrast to H20-A where three slides of each of the manual and automated slide preparations are made and each observer examines separate slides, only one slide was produced for each of the manual and automated slide preparations and the same films were examined by both observers. It is very likely that the majority of the cells examined by the microscopists were the same, so that the cell count was not a total 400 cell count as in H20-A, but rather a duplicate 200 cell count by two independent observers to minimize interobserver variability in cell identification. H20-A provides calculations and tables for the statistical analysis of 200 cell counts and we used them in this study.

### Review of prior papers on automated slidemaker/stainer performance

In the late 1970s, attempts were made to automate the WBC differential count by developing systems that would use computers to process images of cells from blood films in similar fashion to the manual (visual) WBC differential count. Most of these systems required a monolayer blood film with a larger area than that achievable with the manual wedge type smear, so attempts were made to develop automated blood film makers, usually by applying a diluted sample of blood onto a spinning glass slide. A few papers studying the performance of those blood film makers were published at that time (Wenk, 1976; Nourbakhsh *et al.*, 1978; Pierre, 1980). When the image analysis approach to automated WBC differential counts was abandoned, these blood film makers were also abandoned. Approximately 20 years later, systems to automate blood film preparation for visual examination became available, but very few studies of the performance of these modern automated slidemaker/stainers have appeared in independent peer-review journals, or in company journals. Benattar and Flandrin published a paper in 1999 evaluating the performance of a Coulter Slidemaker attached to a Coulter GenS analyzer (Benattar & Flandrin, 1999). In one experiment, they found that the monocyte count from the slidemaker compared better with the monocyte count from a Coulter STKS hematology analyzer (Beckman Coulter Inc.) than manually prepared films. They also found a better correlation coefficient for all normal cell types with the differential count of the STKS for slidemaker films than for manually prepared films. The working area of slidemaker films was larger than manually prepared films. They did not include abnormal cell types in their study.

Descriptions of performance of slidemakers produced by the Sysmex corporation have appeared in a journal published by the Sysmex Corporation; assessments of performance in these papers were qualitative and subjective. In one study by Pawlick and Relopez (2000), manually prepared films from a patient with chronic myelogenous leukemia were compared with films produced by a Sysmex SP-100 automated slidemaker/stainer. Medical technologists in 29 laboratories were of the opinion that the automated slides exhibited overall superior quality and consistency, especially for staining of granules. In another paper published a little later the same year (Bron, 2000), Bron *et al.* wrote that the quality of slides generated by the Sysmex SP-100 automated slide preparation unit using the company supplied settings was less than that of manually prepared slides and that artifacts in white cell morphology and an increase in damaged cells hampered correct interpretation. By making adjustments to prolong the prefix time to 60 s, to change the angle and speed of the spreader slide and to change the settings for spreader slide angle for different hematocrits, they were able to improve the quality of the automated blood films. However, even after the adjustments, the percentage of smudge cells in patients with CLL was always much higher in the SP-100 generated films than the manually prepared films.

### Our results

We studied 139 samples with a wide range of WBC counts and abnormal cells using a rigorous and objective protocol. As described in detail in the Results section, we found that films prepared by automated slidemakers were comparable to well prepared manual films in normal samples as well as those from patients with a number of different diseases and a variety of abnormal cell types in the peripheral blood. All films made by both methods were acceptable for morphological review and differential counting. The distributions of normal white cell types within the counting areas of automated films compared with manual films for all 139 samples, for blasts and promyelocytes in the 65 samples in which they occurred and for NRBCs in the 58 samples in which they occurred fell within the expected limits of 200 cell differential counts as determined by the Standard Error of Proportion calculations and graphs of CLSI H20-A for a range of WBC from 1.0 to 352.8 × 10^9^/l, as shown in Figures 3–9.

Counts for a variety of abnormal cells for proportions as low as 1% were similar on automated and manual films; no sample with abnormal cells was ‘missed’ on any automated or manual film. Smudge cells were observed on both the automated and manual blood films of 19 samples. These were primarily neoplastic samples. In addition, rare or occasional smudge cells were observed on six automated films but not on the manual films in a patient with sepsis, an acute leukemia, a benign lymphocytosis, increased cholesterol and uric acid, increased protein and a normal patient. Although we record in this paper observations on rare and occasional morphological findings, in accordance with the International Consensus Group guidelines for morphological observations on blood films (Barnes *et al.*, 2005), we do not consider them to be significant.

Morphologically significant increased WBC clumps containing four or more cells were produced with similar frequency by the automated and manual systems, as described in detail in the Results section. WBC clumps, defined as two or three cells touching each other, were observed on the sides of the film with similar frequency on the automated slides and the manual slides. WBC clumps of two or three cells were observed in the tail of the film on 40 (28.0%) of the automated slides, including those with the ‘line’ of cells and 23 (16.5%) in the tail of the film of the manual slides. This difference is statistically significant; however, we do not consider clumps of two or three cells to be significant morphologically.

Red cell morphology was comparable between the automated and manual films of all samples and was consistent with the diagnosis. Occasional platelet clumps were observed on both the automated and manual films of three samples.

### Quality control procedures

We could find no prior published work that was comparable in scope to our study of automated slidemaker/stainer performance and it is our impression that the quality control procedures for blood film quality used in our laboratory are unique in scope. Because of the lack of other published work, we are unable to directly compare and assess the effectiveness of our quality control procedures with those of other laboratories. However, it is apparent that these quality control procedures have a major beneficial impact in our laboratory by assuring consistently high quality of blood films produced routinely by four separate slidemaker/stainers as tested in this study on 41 separate days over a period of several months. Samples for this study were selected for the presence of a number of different abnormal cells in a variety of diseases as well as diseases that might interfere with the production of blood films of good quality; a process that required several months. Samples were selected for the study after the sample had been analyzed and the automated blood film had been produced. In no instance was a sample re-run on a slidemaker/stainer to produce a better quality blood film than that obtained when the sample was run for the first time. It would not have been possible to perform the study in this manner unless blood films of good quality were being produced regularly and consistently from all four slidemaker/stainers in routine use.

Our slidemaker/stainers have proved reliable in routine use and we have been able to maintain blood film quality ourselves using the adjustments described in this paper, without requiring field service engineers to perform them. Field service calls were for regular preventive maintenance and for correction of mechanical issues from time to time. Total service calls for 2006, the year in which this study was performed, are detailed in Appendix 2.

We did not perform a side-by-side comparison of automated blood films produced by the slidemaker/stainers of the Beckman Coulter LH755 systems with those of other manufacturers, so we cannot claim that similar adjustments and procedures would produce blood films of similarly high quality on other systems. However, whatever the performance of other slidemaker/stainers may be, we are confident that similar quality control procedures would assure the users of all automated systems that blood films produced by their systems in routine use consistently match the expected performance of those systems.

## Conclusions

When adjusted properly and checked with quality control procedures similar to those applied to laboratory analyzers, automated slidemaker/stainers, as typified by the performance of the four Beckman Coulter LH755 automated slidemakers and stainers in 24 h routine use in our laboratory, are capable of producing and staining blood films on a variety of normal and abnormal samples comparable with well-prepared manual films. No clinical or statistically significant differences were found in distributions of normal white cell types, counts of abnormal cells, cell morphology or blood film artifacts. As the use of automated slidemaker/stainers becomes more widespread, we believe our quality control procedures could be useful to others using these devices; and our approach to evaluation of their performance could give guidance to those who wish to assess the quality of blood films produced by automated slidemaker/stainers of any make or model.

## Appendix 1

### Modified staining procedure

#### Required reagents

- Coulter TruColor Wright-Giemsa stain (2 l).
- Coulter TruColor Wright-Giemsa stain buffer (2 l). Note, this comes as a kit with several small bottles of stain.
- Methanol (99.9%).
- Deionized water.

#### Preparation of stain buffer

Add four bottles (50 ml each from kit) of Wright-Giemsa stain to the 2-l bottle of Buffer. Recap bottle and mix by inversion.

#### Preparation of quick rinse

Add 525 ml methanol to 1575 ml deionized water in reagent plastic bottle and mix well.

#### Preparation of methanol-stain mixture

Add 20 ml of TruColor Wright-Giemsa Stain to 2 l of Methanol in reagent plastic bottle and mix well. Note volume can be adjusted based on workload. Slide baskets should be properly dried before being used in the staining process to prevent contamination. This mixture can be modified per user as to the volume of stain.

**Table:** Staining procedure for Beckman Coulter LH755 slide stainer

Baths	Reagents	Preparation	Time
#1	Methanol-stain	2 l methanol + 20 ml stain	20 s
#2	Wright-Giemsa	Stock/as delivered	2 min
#3	Buffered-Giemsa	2 l buffer + 200 ml stain	3 min
#4	Quick rinse	525 ml methanol + 1575 ml water	10 s
#5	Deionizedwater	Supply source	1 min 30 s
Dryer			4 min

## Appendix 2

### Field service calls

Each of the four slidemaker stainers has two preventive maintenance visits per year from field service engineers and the dispense tubing is replaced four times a year.

**Table:** Total unscheduled service calls for repair in 2006:

Slidemaker #1
Slide basket movement error (1)
Slide ejector error (1)
Slidemaker #2
Slide ejector error (3)
Printer problems (2)
Slidemaker #3
Basket transfer problems (3)
Probe problem (1)
Vacuum sensor problem (1)
Tubing replacement (1)
Slidemaker #4
Vacuum sensor problem (3)
Printer problem (2)
Slide dryer problem (1)
Leak in the slide access module (1)

Most problems were corrected the same day within 2–4 h. On two occasions, parts had to be ordered and were delivered the following day.
